# Discontinuation of implants use and associated factors among women attending health facility clinics in Hawassa City, Southern Ethiopia, 2019; cross sectional study

**DOI:** 10.1186/s40834-020-00128-3

**Published:** 2020-10-23

**Authors:** Belay Amare Abebe, Mulu Reda Terefe

**Affiliations:** 1 Department of Midwifery, College Medicine and Health Sciences, University of Hawassa, PO Box-1560, Hawassa, Ethiopia; 2 Department Statistics, College of Natural and Computational Science, University of Hawassa, PO Box-1560, Hawassa, Ethiopia

**Keywords:** Associative factors, Discontinuation, Implants

## Abstract

**Background:**

Despite improving the availability and use of Implants, discontinuation is becoming a public health concern. A significant proportion of women discontinuing the service before its due date, which is of concern in the health system and its consequence may lead to a program failure. This might have also social and economic consequences for users. Only 8% of married women in Ethiopia use implants. Apart from its low utilization, premature removal is common for unknown reasons. However, there is paucity of information on discontinuation of implants use and associated factors in the study area.

**Objective:**

The study was aimed to assess discontinuation of implants use and associated factors among women attending health facility clinics in Hawassa city, southern Ethiopia, from March, 01-April, 01/2019.

**Methods:**

Facility based cross sectional study design was used. Out of 16 health facilities, 9 of them were selected for this study using simple random sampling. Total sample size of this study was determined to be 351. Data were collected from study subjects using pretested, structured questionnaire through a face-to-face interview. Data was analyzed using descriptive statistics and logistic regression. The result is presented using the Crude Odds ratio as well as Adjusted Odds Ratio with the corresponding 95% confidence level.

**Result:**

Out of 351 study participants, the overall proportion of implants discontinuation was 49.3%(95% CI: 44.2–55.0). Lack of counseling about side effects (AOR = 2.394; 95% CI: 1.422–4.030), developing side effects (AOR = 6.325; 95% CI: 3.719–10.757) and lack of post insertion follow-up (AOR = 2.241; 95% CI: 1.186–4.234) the major factors associated with discontinuation of Implants.

**Conclusion and recommendation:**

In this study, the overall proportion of discontinuation of Implants among women who were using Implants was high. Health professionals could give pre-insertion counseling about side effects and post insertion dates for follow-up to improve of utilization of implants.

## Backround

Modern contraception is highly effective in preventing unintended pregnancy and reducing maternal mortality. Family planning (FP) is a process that usually involves a discussion between a woman, a man, and a trained family planning service provider focusing on family health and the desires of the couple to either limit or space their family size [1]. Contraceptives discontinuation is removal of the methods by the women due to any reason. Implants are long-acting and extremely effective at preventing pregnancy, with a clinical failure rate less than 1% [19].

Discontinuation of effective methods of contraception is a universal problem, though rates vary widely by population and country [8]. Every year, about one-third of the 182 million pregnancies occurring worldwide are unplanned [7, 21]. Globally, 33 million accidental pregnancies are estimated to occur among women reportedly using a contraceptive method, either traditional or modern [25].

Evidence from 60 demographic and health surveys conducted on causes and consequences of contraceptive discontinuation; on average, 38% of women discontinue using reversible methods by the 12th month and 64% by the 36th month in 19 countries [25].

According to a report based on developing countries, Implanon discontinuation within the first year ranges from 2% in Nigeria to 23% in UK [2, 3].

Contraceptive discontinuation contributes to unplanned pregnancies, unwanted births and termination of pregnancies that expose a risk to the health of women. This unplanned pregnancy has impacts on larger family size and ultimately contributes to higher overall fertility rates which may result social, economic and physical health disabilities [18, 23].

According to [11], contraceptive discontinuation in the 5 years preceding the survey were > 35% within 12 months’ use [11].

Only 8% of married women in Ethiopia use implants [12, 26]. Apart from its low utilization, premature removal is common for unknown reasons. In addition, there is limited information on discontinuation of implants use and associated factors in the study area. Therefore, this study was aimed to assess discontinuation of implants use and associated factors among women attending health facility clinics in Hawassa city, Southern Ethiopia, from March, 01 to April, 01/2019.

The findings of this study will enhance the planning and decision-making capacity of health professionals to seek possible solutions to the problems of the community in collaboration with the stake holders concerned. In addition, the findings will help local programmer managers, planners and other concerning organizations working in the field of family planning and maternal health to plan new strategies and prepare training programs based on the identified factors to enhance retention of utilization in the community. Finally, this study will provide baseline data for other researchers to investigate a prospective future study in the field.

### Conceptual framework (Fig. 1)

**Fig. 1:**
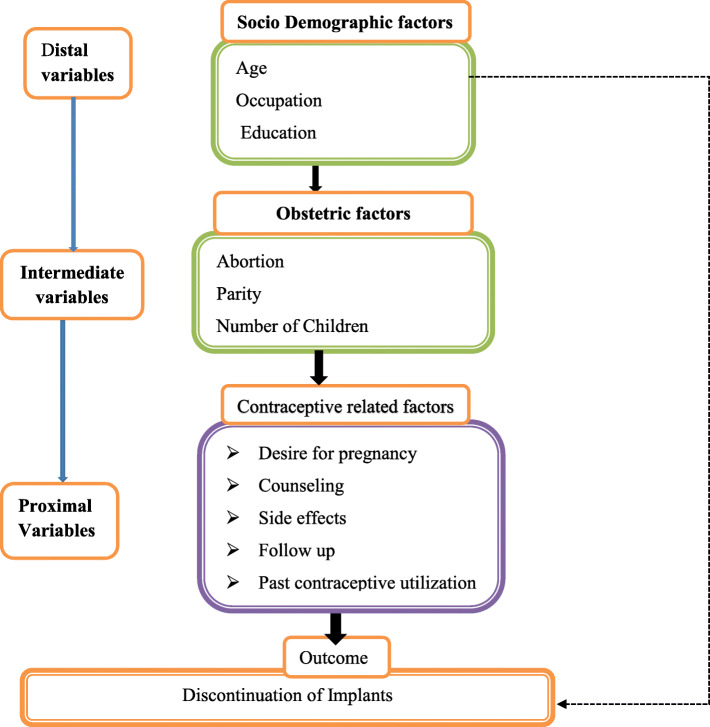
Conceptual framework for the study on assessment of discontinuation of Implants and associated factors developed by principal investigator. This conceptual framework is developed from different literature used from a range of cross sectional and case control studies conducted on early discontinuation of use of Implanon and associated factors in Ethiopia [6, 15]

## Methods and materials

### Study area and period

Hawassa City Administration, is the capital city of Southern nation nationality people, and Sidama Zonal administration, located 275 km to the south of the capital city of Ethiopia, Addis Ababa. The city administration is divided in 8 sub cities, of which 7 are urban having 21 kebeles and one peri-urban with 12 kebeles with a total population of 387,087 in 2017 (CSA, 2017). The study was conducted in nine health facilities, i.e. HURH, AGH, Hawassa family guidance association, Hawassa Merry stop international clinic, Millinium HC, Alamura HC, Adare HC, Tulla HC and Tilite HC of Hawassa City, Southern Ethiopia, from March, 01 to April 01/2019.

### Study design

Facility based cross-sectional study was used.

### Study population

All reproductive age women who were using Implants and came to the selected health institutions for contraceptives related reasons during the actual data collection period.

### Inclusion and exclusion criterias

All reproductive age women who were using Implants and came to the selected health institutions for contraceptives related reasons during the actual data collection period were included in the study. Women who were unable to communicate and respond like hearing loss were excluded from the study.

### Sample size determination

Outcome variable and various factors significantly associated with the outcome variable in previous studies were considered to determine the sample size. Accordingly, for the first and second specific objectives the sample size was calculated separately and the larger sample size was taken to be used for this study.

### Specific objective 1

For magnitude of discontinuation of Implants.

The sample size was calculated by using single population proportion formula as follows.

$$ \boldsymbol{n}=\frac{{\left({\boldsymbol{z}}_{\boldsymbol{\alpha} /\mathbf{2}}\right)}^{\mathbf{2}}\times \boldsymbol{p}\left(\mathbf{1}-\boldsymbol{p}\right)}{{\boldsymbol{d}}^{\mathbf{2}}} $$

Where *n* is the minimum sample size required, *p* is expected proportion of discontinued mothers, **z =** 1.96 (95% CI) and *d* = 0.05 is the margin of error between the sample and the population. For this study *p* = 16%, from study conducted on Ofla Tigray on the prevalence of discontinuation of implants and associated factors. Thus, applying the formula, by substituting these values into Eq. (1) gives, = 206.5. By adding 10% non-response rate, the final sample size becomes 227 for the first specific objective.

### Specific objective 2

factors associated with discontinuation of Implants.

The sample size of the second specific objective of this study was calculated by considering factors that were significantly associated with the outcome variable, two sided confidence level of 95%, the margin of error of 5%, power by 80% and the ratio of exposed to unexposed 1:1 using Epi-Info Version 7 software.

As it can be seen from Table 1, the calculated sample size of the second objective is larger than that of the first objective. By adding 10% non-response rate, *n* = 313*0.1 + 313 = 351

**Table 1:** Sample size determination for magnitude and associated factors of Implants discontinuation among Implants user women in health facilities of Hawassa city, Southern Ethiopia, 2018/19

Associated factors	Exposed outcome %	Unexposed outcome %	Sample size	Reference
Removal due to side effect	75.6(side effect faced)	60.2(side effect not faced)	313	[15]
Follow up	35.9(had follow up)	15.76(hadn’t follow up)	166	[22]

### Sampling technique / procedure

Out of the 16 health facilities available in Hawassa city administration, both hospitals (i.e. HURH and Adare General Hospital), five HCs, (i.e. Millinium, Adare, Tilite, Tulla and Alamura HCs) and two NGO clinics (i.e. Hawassa family guidance association and Hawassa Marie stops international) were randomly selected for this study using lottery method.

In order to ensure a proportionate allocation of the total sample size determined, the average six-month client flows of each selected health facility were considered prior to the start of data collection. The study subjects were identified based on the information obtained from the client card. The information obtained from the 6 Months enrollment record (June–December/2018) from the family planning registry books showed that a total of 3500 women were used Implants within 6 months in the selected institutions.

As the result, the average six-months client flow for HURH, AGH, Hawassa family guidance association, Hawassa Marie stops international clinic, Millinium HC, Alamura HC, Adare HC, Tulla HC and Tilite HC was 500, 560, 440, 400, 310, 380, 300, 360, and 250 respectively.

**Fig. 2:**
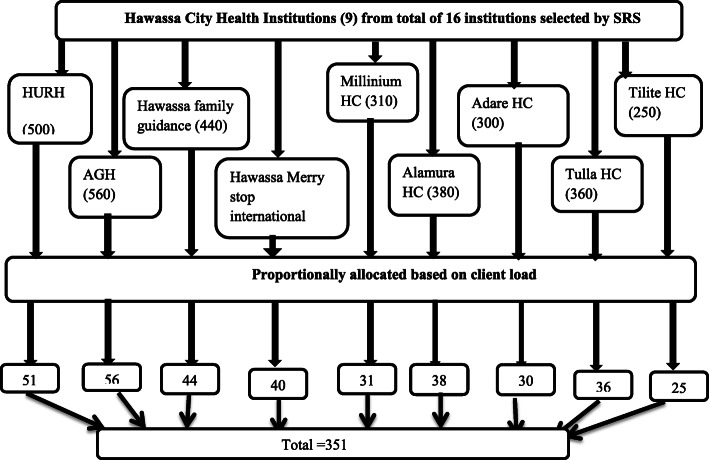
Schematic presentation of sampling procedure for the study on discontinuation of implants and associated factors developed by principal investigator. Six months’ enrollment in Heath institutions of Hawassa city administration in both public hospitals, five HCs and two NGO clinics from family planning registration book, for sample selection (June–December/2018)

So that, sample was proportionally allocated for each selected institution. Data collectors approached and recruited implants user women who came to the selected clinics due to contraceptives related reasons. Every woman was included in the study until the required sample size was met in each health facility (Fig. 2).

## Data collection methods

### Data collection instruments

The data for this study was collected using pre-tested, structured questionnaire through a face to face interview. The tool for this study is adopted from previous literature of similar studies [6, 15]. The questionnaire contains three main parts. These are; socio-demographic characteristics, Obstetric related factors and contraceptive methods related variables.

### Data collectors

Nine Nurses and Midwives with a qualification of degree were the data collectors. Three supervisors supervised and provided all items necessary for data collection on each data collection day for data collectors.

### Data collection procedure

Data were collected from study subjects using pretested, structured questionnaire through a face-to-face interview. The questionnaire was developed in English and translated into Amharic and then back to English to check for its consistency. The instrument was adopted from different literature developed for similar purposes by different authors [6, 15]. The interviewers were Midwives and Nurses with a qualification of degree. The responsibility of the data collectors was to fill the questionnaire after obtaining informed, voluntary, written and signed consent of the study subjects. The principal investigator and supervisors supervised and provided all items necessary for data collection on each data collection day, checking filled questionnaire for completeness and logical consistency and solve problems forwarded during the time of data collection.

### Data quality assurance

To assure the quality of data, properly designed data collection tools were used. Training was given for data collectors and supervisors about research objectives, data collection tools, procedures and interview techniques for 1 day. The principal investigator, together with three supervisors, supervised techniques of data collection and completeness of the tools on the daily basis to give an appropriate feedback accordingly. Data double entry was done to make comparisons of two data clerks and resolved some difference.

### Pre-test

Before the actual data collection period, the questionnaire was tested on 5% of the total sample size (351) on 18 reproductive age women who used implants at none-selected health facilities to check the context of data. After pretest, the questionnaire was revised and amended per necessary.

#### Data processing and analysis

After data collection, the questionnaire was checked for completeness and data entry was made using EPI DATA V-3.1 and then exported to the Statistical Package for Social Science [SPSS] V-22 computer software for analysis. Descriptive statistics and bivariate analysis were performed. Variables with *p*-value < 0.25 in bivariate analysis were planned to be entered in multivariate logistic regression analysis. Multi co-linearity test was carried out to check the linear correlation among independent variables by using standard error and co-linearity statistics. Variance inflation factor > 10 and standard error > 2 were considered as suggestive of the existence of multi co-linearity. Therefore, variables with Variance inflation factor > 10 and/ standard error > 2 were checked to be dropped. Hosmer -Lemeshow goodness- of- fit was used to check model fitness. Omnibus test was significant and Hosmer- Lemeshow’s test was found to be insignificant which indicate that the model was fitted. Based on the findings of bivariate analysis, variables with *p*-value< 0.25 were entered into multivariate logistic regression analysis with 95% confidence level and 5% significant level. AOR with 95%CI, and level of significant at *p*-value < 0.05 was considered. Variables with *p*-value less than 0.05 in the multivariate logistic regression analysis were considered as statistically significant with implants discontinuation.

## Result

### Socio-demographic characteristics

All study participants had responded to the questionnaire, making a response rate of 100%. The range of study participants’ age was between 19 and 44 years with mean (±SD) age 26.6 ± 4.3. Out of the total respondents, 164 (46.7%) of women were in the age range of 25–29. Three hundred forty three (97.7%) of participants were married and about 218 (62.1%) were Protestant followed by Orthodox, 103 (29. 3%) in religion. One hundred forty six (41.6%), of participants were Sidama in Ethnicity.

Among the participants, 149 (42.5%) were housewives and 166 (38.5%) of their husbands were merchants by occupation. Ninety nine (28.2%) of the participants were at primary level and 141 (41.1%) of their husbands were college and above in educational status (Table 2).

**Table 2:** Socio-demographic characteristics of the study participants in health facilities of Hawassa city, Southern Ethiopia, 2019 (*n* = 351)

Variables	Frequency	Percentage
**Women’s age**		
19–24	100	28.5
25–29	164	46.7
30–34	69	19.7
= > 35	18	5.1
**Marital status**		
Single	8	2.3
Married	343	97.7
**Religion**		
Orthodox	103	29.3
Muslim	20	5.7
Protestant	218	62.1
Catholic	9	2.6
Others	1	3
**Ethnicity**		
Sidama	146	41.6
Wolayta	82	23.4
Amhara	62	17.7
Gurage	25	7.1
Oromo	14	4.0
Others*	22	6.3
**Occupation**		
House wife	149	42.5
Merchant	62	17.7
Private employee	47	13.4
Government employee	65	18.5
Student	28	8.0
**Maternal educational status**		
Unable to read and write	44	12.5
Read and Write	39	11.1
Primary	99	28.2
Secondary	82	23.4
College and above	87	24.8
**Husband educational status (*n*** **= 343)**		
Unable to read and write	24	7.0
Read and Write	23	6.7
Primary	55	16.0
Secondary	100	29.2
College and above	141	41.1
**Husband’s occupation (*n* = 343)**		
Farmer	21	6.1
Merchant	123	35.9
Private employee	59	17.2
Government employee	123	35.9
Student	7	2.0
Others	10	2.9

### Obstetrics related characteristics

Two hundred and seven (59.0%) respondents had given birth one to two times while 116 (33.0%) of the participants gave birth more than three times. Among the participants who gave birth, 226 (64.4%) women had one to two alive children. Two hundred and forty-four (69.5%) of participants had no history of abortion and 239 (68.1%) women had desire for another pregnancy. Out of those who had desire for another pregnancy, 94 (39.3%) and 145 (60.9%) women desired to become pregnant within two and after two years respectively (Table 3).

**Table 3:** Obstetrics related factors for discontinuation of implants among implants user women in health facilities of Hawassa city, Southern Ethiopia, 2019 (*n* = 351)

Variables	Frequency	Percentage
**Parity**		
0	28	8.0
1–2	207	59.0
3+	116	33.0
**Number of living children**		
0	29	8.3
1–2	226	64.4
3–4	86	24.5
5+	10	2.8
**History of abortion**		
Yes	107	30.5
No	244	69.5
**Desire for pregnancy**		
Yes	239	68.1
No	112	31.9
**When they want to become pregnant(*n*** **= 239)**		
Within two years	94	39.3
After two years	145	60.7

### Contraceptive related characteristics

In this study, 261 (74.4%) and 90 (25.6%) of participants were Implanon and Jaddle users respectively. Three hundred and thirty (65.5%) of study participants had ever used contraceptives before the implant they were using during the study period. Out of them 176 (76.5%) had used injectable followed by oral contraceptive pills 83 (36.1%). Of the total participants, 305(87%) women got the implant insertion from government institutions and 252 (71.8%) of them chose the contraceptives by their own.

Three hundred and twenty-seven (93.2%) and 165 (47.0%) of the participants got counseling service about the benefit and side effect of implants during insertion respectively. Two hundred and seventy-five (78.3%) of study participants had an appointment for follow-up during their implant utilization period. One hundred and thirty-six (38.7%) of the study participants had ever faced side effects during their implants utilization period. The suggested reasons for discontinuation of implants were identified that side effect was the major, which accounted 79 (45.7%) followed by 41 (23.7%) of method switching after service end (Table 4).

**Table 4:** Contraceptives related characteristics for discontinuation of implants in Health facilities of Hawassa city, Southern Ethiopia, 2019 (*n* = 351)

Variables	Frequency	Percentage
**Type of implant used by participants**		
Implanon	261	74.4
Jaddle	90	25.6
**Ever used contraceptives before implant**		
Yes	230	65.5
No	121	34.5
**Type of contraceptive used before implant**		
OCP	83	36.1
Injectable	176	76.5
IUCD	4	1.7
Implants	34	14.8
**Place of insertion of the method**		
Hospital	162	46.2
Health center	141	40.2
Health post	2	0.6
Family guidance	40	11.3
Others*	6	1.7
**Counseling for benefit of implants**		
Yes	327	93.2
No	24	6.8
**Counseling for side effects of implants**		
Yes	165	47.0
No	186	53.0
**Estimation of information participants get during counseling (satisfaction)**		
Yes	272	77.5
No	79	22.5
**Have you ever faced side effects**		
Yes	136	38.7
No	215	61.3
**Who choose implant**		
Own choice	252	71.8
My husband	31	8.8
Health professional	65	18.5
Health extension	2	0.6
My neighbor	1	0.3
**Reasons to choose the method**		
Safety	70	19.9
Long protection	150	42.7
It can remove at any time	86	24.5
Immediate fertility return	45	12.8
**Follow-up**		
Yes	275	78.3
No	76	21.7
**Removal due to side effec**t(*n* = 221)		
Yes	79	35.7
No	142	64.3

Others* Merry stopes international clinic, private hospitals and clinics

**Fig. 3:**
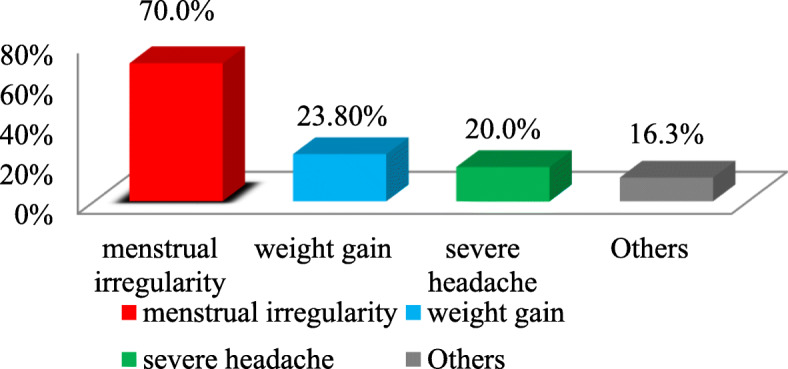
The main side effects for discontinuation of implants among implants user women in health facilities of Hawassa city administration, Southern Ethiopia, 2019. Others*: Insertion arm pain, achene, weight reduction and difficult to work

Among participants who discontinued implants due to non-side effect reasons, method switching after service accounted 41 (23.7%) followed by desire for another pregnancy, 40 (23.1%) and others like husband go abroad, husband’s objection, divorce and health concern accounted about 13 (7.5%). Whereas, among women who discontinued implants due to side effects, menstrual disruption accounted 56 (70.0%) followed by 19 (23.8%) weight gain (Fig. 3).

### Magnitude of implants discontinuation

**Fig. 4:**
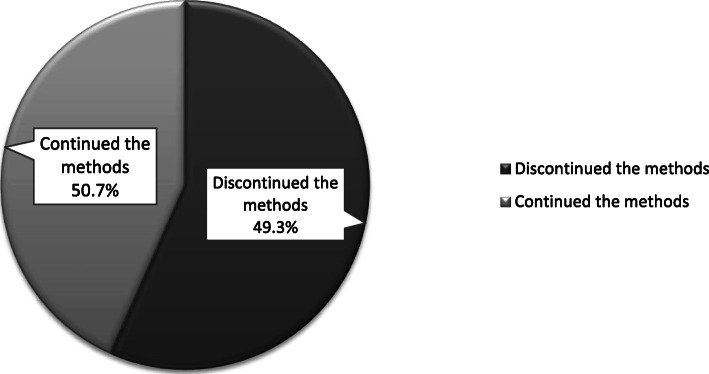
Proportion of implants discontinuation among implants user women in health facilities of Hawassa city administration, Southern Ethiopia, 2019

From 351 study participants, 49.3% (95% CI: 44.2, 55.0%) was overall proportion of Implants discontinuation **(**Fig. 4**)**. The mean (±SD) overall duration of Implants utilization in years was 2.2 ± 1.1.

### Factors associated with implants discontinuation

Findings from bivariate analysis resulted that, participants’ occupational status, maternal educational status, Husband educational status, past contraceptive utilization, lack of counseling about possible benefits, lack of counseling about side effects, lack of appointment for follow-up and developing side effects were associated with discontinuation of implants at *p*-value< 0.25. However, in a multivariate logistic regression analysis, lack of counseling about side effects (AOR = 2.394; 95% CI: 1.422–4.030), lack of appointment for follow up (AOR = 2.241; 95% CI: 1.186–4.234) and developing side effects (AOR = 6.325; 95% CI: 3.719–10.757) were found significantly associated with implants discontinuation (Table 5).

**Table 5:** Factors associated with implants discontinuation among implants user women in health facilities of Hawassa city, Southern Ethiopia, 2019 (*n* = 351)

Variables	LARCs discontinuation Ye s (%) No (%)		***P***-value	AOR (95% C.I)
**Occupation of participant**				
House wife	71 (47.7%)	78 (52.3%)	**0.110**	2.622 (0.855–8.041)
Merchant	30 (48.4%)	32 (51.6%)	**0.164**	2.613 (0.801–8.529)
Private employee	27 (57.4%)	20 (42.6%)	0.559	1.531 (0.456–5.141)
Government employee	32 (49.2%)	33 (50.8%)	**0.184**	2.337 (0.71–7.694)
Student	18 (64.3%)	10 (35.7%)		1
**Maternal Educational status**				
Unable to read and write	22 (50.0%)	22 (50.0%)	0.663	1.423 (0.360–5.633)
Read and Write	23 (59.0%)	16 (41.0%)	0.605	0.798 (0.235–2.712)
Primary	42 (42.4%)	57 (57.6%)	**0.115**	2.240 (0.793–6.329)
Secondary	44 (53.7%)	38 (46.3%)	0.962	1.095 (0.427–2.811)
College and above	47 (54.0%)	40 (46.0%)		1
**Husband‘s educational leve**l				
Unable to read and write	13 (48.1%)	14 (51.9%)	0.708	0.581 (0.159–2.1177)
Read and Write	15 (60.0%)	10 (40.0%)	0.465	0.351 (0.112–1.118)
Primary	31 (56.4%)	24 (43.6%)	0.589	0.446 (0.189–1.052)
Secondary	44 (44.0%)	56 (56.0%)	**0.215**	0.903 (0.444–1.836)
College and above	75 (52.1%)	69 (47.9%)		1
**Ever used contraceptives before implants**				
Yes	106 (46.1%)	124 (53.9%)	**0.017**	1.613 (0.940–2.769)
No	72 (59.5%)	49 (40.5%)		1
**Counseling about side effects of implants**				
Yes	108 (65.5%)	57 (34.5%)	**< 0.0001**	2.394 (1.422–4.03) **
No	70 (37.6%)	116 (62.4%)		1
**Counseling about benefit of implants**				
Yes	171 (52.3%)	156 (47.7%)		1
No	7 (29.2%)	17 (70.8%)	**0.034**	2.312 (0.825–6.480)
**Side effects**				
Yes	34 (25.0%)	102 (75.0%)	**< 0.0001**	6.33 (3.719–10.76) ***
No	144 (67.0%)	71 (33.0%)		1
**Follow-up**				
Yes	155 (56.4%)	120 (43.6%)		1
No	23 (30.3%)	53 (69.7%)	**< 0.0001**	2.241 (1.186–4.234) *

Significant at ***p < 0.0001, **p = 0.001, *p = 0.013, *AOR* Adjusted odds ratio

## Discussion

The proportion of implants discontinuation among women who were users of implants was high. Factors such as lack of counseling about side effects, developing side effects and lack of appointment for follow up were significantly associated with discontinuation of implants.

The proportion of implants discontinuation among implants user women was 49.3%. This finding is in line with studies conducted in Netherland, 47% [23], Jordan, 45%(USAID, 2007–2009) and Cambodia, 45% [21]. However, the current proportion is lower than study conducted in Australia, 60% [13] and at Debre Tabor 65% [15]. This result variation might be due to study nature of study participants. In studies conducted in Australia and Debre Tabor, the study participants were both urban and rural, but urban only in this study. As a result, awareness towards the behaviors of contraceptives might be better at urban residents. Moreover, it might be due to efforts made by health providers to improve the retention of utilization.

The current proportion is also higher than studies conducted in the United States, 25.2% [10], Pakistan, 18.0%, Egypt, 36%, Yemen, 43%, [21], Ilorin, Nigeria, 26.5% [4], Abakaliki, southeast Nigeria, 29.6% [14] and at Ofla Tigray, 16%, [6]. The difference might be due to lack of pre-insertion counseling of side effects of the methods as compared to other studies (USAID, 2007–2009). In addition to this, it might be due to lack of appointment for follow up as compared to other studies [4, 10, 21, 23]. The other reason for differences in current proportion might be due to higher sample size of the current study compared to other studies [6, 10, 14]. Another possible reason might be due to sociocultural differences of respondents across the study areas.

This study showed that women who didn’t get pre-insertion counseling service about side effects were 2.4 times more likely to discontinue than those who got counseling services. This is consistent with study conducted in Tigray [6], Debre Tabor [15], Jordan, India [5], Diguna Fango [22] and Philippines [20]. Providing counseling about side effects of implants was positively associated with use of the methods [20, 22]. So, the possible justification of this factor might be due to lack of pre-placement counseling and support by the service providers to help women continue on contraception. In addition, sociocultural variations, lack of skilled counselor, outlook and level of understanding of participants towards the contraceptives might be the reasons that made the factor associated with discontinuation of the implants.

According to this study, the likelihood of discontinuing implants among women who developed side effects was 6.3 times higher than those who did not face side effect. This is consistent with studies conducted in Ankara, Turkey, Abakaliki, southeast Nigeria [14], Debre Tabor [15], Arsi, Oromia region [9], Jos, Nigeria [16], Tigray [6], Nepal [24] and Jordan. The possible reasons might be exposing to side effect of the method could contribute for discontinuation of the contraceptives. In addition, respondents who discontinued implants due to side effects may be due to intolerance of side effects and fears that different complications will occur in their health in the future.

This study also revealed that, the odds of discontinuation of implants among women who lacked post insertion follow up were 2.2 times higher than those who had appointment for follow up. This is in line with studies conducted in Port, Nigeria [17] and Diguna Fango [22]. It might be due to the fact that post insertion follow-up increases retention of utilization, client trust on health providers, and knowledge about the overall behaviors of the contraceptives via participants.

This study includes nine health facilities (multicenter study) which increase external validity of the study.

Recall bias was not challenging unlike studies conducted on ever used participants.

The study was conducted in an urban health institution setting, though the majority of the population lives in rural setting.

## Conclusion

In this study, the overall proportion of discontinuation of implants was high (49.3%). Out of the overall proportion of discontinuation, reason of side effects and husband objection accounted 23.4%. Factors like lack of counseling about side effects, developing side effects and lack of post insertion follow-up were found statistically significant with implants discontinuation.

### Strengths

Recall bias was not challenging unlike studies conducted on ever used participants.

### Limitation

The study was conducted in an urban health institution setting, though the majority of the population lives in rural setting.

### Recommendations

Health organizations and other stake holders better to work on health care providers at enabling them to give appropriate pre-insertion counseling and post insertion date of follow-up in order to increase the retention of utilization.

Health professionals could give pre-insertion counseling with giving emphasis on possible side effects of implants.

Early side effects management and reassurance is recommended to decrease discontinuation and improve the retention of utilization of implants.

In addition to this, health providers could give post insertion date of follow-up for their clients.

## Data Availability

Data and materials are fully available.

## Abbrivations

AOR: Adjusted odds ratio
BDHS: Bangladesh Demographic Health Survey
COR: Crude odds ratio
CSA: Central Statistical Agency
EDHS: Ethiopia Demographic Health Survey
EFMOH: Ethiopian Federal Ministry of Health
FHI: Family Health International
HURH: Hawassa University Referral Hospital
ICF: International classification of functioning, disability and health
IUCD: Intra uterine contraceptive device
JPFHS: Jordan Population and Family Health survey
MDGs: Millinium development goals
OPDs: Out- Patient Department
SNNPRS: Southern Nation Nationality People’s Representative state
SDG: Sustainable development goals
UK: United Kingdom
USAID: United States Agency of International Development
WHO: World Health Organization
